# Causal Relationships between Homocysteine and the Polycystic Ovary Syndrome: A Mendelian Randomization Analysis

**DOI:** 10.1155/2024/3090797

**Published:** 2024-05-07

**Authors:** Xianping Lin, Yaojuan Jin, Shihao Hong

**Affiliations:** ^1^Nursing Department, Sir Run Run Shaw Hospital, School of Medicine, Zhejiang University, Hangzhou 310016, China; ^2^Hospital Department of Obstetrics and Gynecology, Linhai Second People's Hospital, Taizhou 317016, China; ^3^Department of Obstetrics and Gynecology, Sir Run Run Shaw Hospital, School of Medicine, Zhejiang University, Key Laboratory of Reproductive Dysfunction Management of Zhejiang Province, Hangzhou 310016, China

## Abstract

**Background:**

Polycystic ovary syndrome (PCOS) is an endocrine disease attributed to multiple genetic variants and environmental factors. We aimed to find the causal association of homocysteine (Hcy) with PCOS.

**Methods:**

A two-sample Mendelian randomization (MR) analysis was performed. We selected 14 single-nucleotide polymorphisms (SNPs) as instrumental variables to predict the risk of PCOS from genome-wide association studies (GWAS). The summary statistics of PCOS were obtained from 3 large genome-wide association studies in the European population, involving 4,138 cases and 20,129 controls, 3,609 cases and 229,788 controls, 994 cases and 165,817 controls, separately.

**Results:**

The IVM analyses revealed that plasma Hcy levels were not causally associated with the risk of PCOS in the meta-analysis (combined effect = 1.032, 95% confidence interval (CI): 0.885–1.203, *p*=0.688).

**Conclusions:**

There was no sufficient evidence to support the causal association of the Hcy with the risk of PCOS.

## 1. Introduction

Polycystic ovary syndrome (PCOS) is a commonly encountered endocrinopathy in women of reproductive age, arising from the interaction between multiple genetic variants and environmental factors [1]. At present, there are three distinct diagnostic criteria for PCOS, namely, the National Institutes of Health (NIH) criteria, the Rotterdam criteria, and the Androgen Excess and PCOS (AE-PCOS) Society criteria, each with a prevalence of 6%, 10%, and 10% for PCOS, respectively [2–4]. The Rotterdam diagnostic criteria, predominantly adopted by experts worldwide, requires the presence of two out of three traits: [1] oligo- and/or anovulation, [2] hyperandrogenism, and [3] polycystic ovaries [3, 5]. PCOS is associated with an elevated risk of developing obesity, metabolic syndrome, type 2 diabetes, and cardiovascular disease [6].

Homocysteine (Hcy), an intermediary amino acid formed during methionine metabolism, is metabolized by remethylation into methionine or by transsulfuration into cysteine [7]. Disruption of these metabolic pathways results in hyperhomocysteinemia, influenced by genetic factors such as MTHFR polymorphism and vitamin deficiencies such as folate, vitamin B6, or vitamin B12 [8]. Emerging evidence suggests that an elevated plasma Hcy level is a risk factor for cardiovascular disease, stroke, obesity, and diabetes [9–12]. At the same time, numerous studies have documented that hyperhomocysteinemia is associated with PCOS [13–22], although inconsistent findings have also been reported in other studies [23–27]. Nevertheless, the interpretation of these observational studies is limited by confounding variables (e.g., age, BMI, and smoking status) and reverse-causality bias.

**Figure 1:**
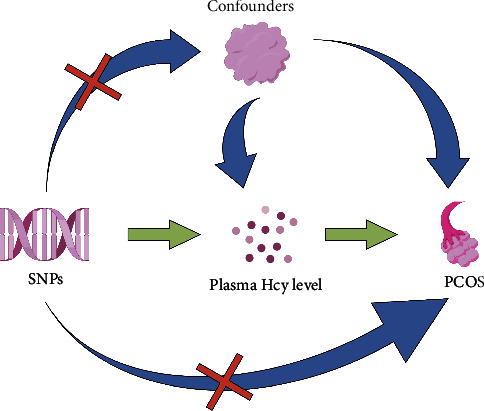
Study design of the Mendelian randomization analysis between homocysteine and the risk of polycystic ovary syndrome. PCOS: polycystic ovary syndrome and Hcy: homocysteine. The “×” means our study design avoids such a situation.

To gain deeper insights into the nature of the observed associations, Mendelian randomization (MR) is recommended for advanced study, utilizing genetic variations as instrumental variables (IVs) for exposures. MR is an evolving methodological approach that employs IVs, including single-nucleotide polymorphisms (SNPs) identified through genome-wide association studies (GWAS), to delineate causal relationships between exposures and disease outcomes. It is less susceptible to the aforementioned shortcomings, given that genetic variations are randomly allocated and remain steady throughout life, owing to Mendel's first and second laws of heritability [28, 29]. However, MR studies rely on the following assumptions: [1] selected IVs must be reliably associated with the exposure factor; [2] selected IVs can only affect the outcome through the exposure; and [3] selected IVs cannot be associated with confounding factors. In recent years, MR methods have been widely applied to elucidate the etiology and consequences of PCOS using GWAS data [30], but no relevant studies for Hcy and PCOS have been undertaken so far. In the current study, a two-sample MR was performed to evaluate the causal role of hyperhomocysteinemia on PCOS using SNPs as IVs, Hcy as the exposure, and PCOS as the outcome in European populations. A brief overview of the MR study design is illustrated in Figure 1.

## 2. Materials and Methods

### 2.1. IVs Selection

We obtained instrumental variables (IVs) from a genome-wide association study (GWAS) comprising 10 European population cohorts with a total of 44,147 white individuals [31] and 18 SNPs from 13 loci were found to be associated with plasma Hcy levels at a genome-wide significance (*p* < 5 × 10^−8^). Using the European population of 1000 Genomes as a reference panel, linkage disequilibrium was estimated across SNPs (*r*^2^ < 0.05), and three SNPs (rs12134663, rs957140, and rs12921383) were removed from our study to make the remaining SNPs independent. To ensure that the specified SNPs only influenced the outcome through the exposure, we used the PhenoScanner V2 website (https://www.phenoscanner.medschl.cam.ac.uk) to kick out the SNP associated with any potential confounders of PCOS and rs548987 was removed because of pleiotropic effect on BMI. Three [32] SNPs (rs7422339, rs234709, and rs2851391) were not available in the FinnGen datasets and did not get a replacement with proxy SNPs (*r*^2^ > 0.8). Lastly, a total of 14 (11 in FinnGen) SNPs were selected (Table 1), explaining 5.9% of the variation in the Hcy plasma levels [31], and these SNPs were selected by the previous studies to predict the serum level of Hcy [32–34]. In advance, the F statistic was calculated to detect the strength of the IVs, and *F* < 10 meant a weak IV scenario [35].

### 2.2. Study Outcomes

Genetic associations with PCOS were gathered from 3 large genome-wide association studies (GWAS) in the European population. The study conducted by Day et al. included 4,138 cases of PCOS based on NIH or Rotterdam criteria and 20,129 controls collected from six cohorts (self-report-based data from 23andMe was excluded) [36]. Using the ICD code of PCOS (ICD-10 code E28.2, ICD-9 code 256.4, or ICD-8 code 256.90), the study performed by Tyrmi et al. comprised 3,609 cases and 229,788 controls from Finland and Estonia [37], whilst the FinnGen study release 7 (R7) consisted of 994 cases and 165,817 controls (https://finngen.gitbook.io/documentation/v/r7/) [38]. A description of all data sources is summarized in Table 1. Written consent was provided by participants, and the relevant ethical review boards approved all the studies involved in the MR analysis. Considering that the data used herein were previously published, ethics committee approval was waived.

### 2.3. Statistical Analyses

The inverse-variance-weighted (IVW) method was applied to evaluate the association between plasma Hcy levels and the risk of PCOS. A *p* value of <0.05 was considered a statistically significant difference [39]. For sensitive analysis, the weighted median estimator, which can yield consistent results even when half of the instrumental variables are invalid, was used [40]. The MR-Egger regression method and the MR-PRESSO method were also used to evaluate the directional pleiotropy by calculating the intercept of the association between Hcy and PCOS [41]. The MR-Steiger filtering was used to estimate potential reverse causal impact.

Leave-one-out sensitivity analysis was performed for the heterogeneity test to explore the influence of specific SNPs on the association. All statistical analyses were conducted using the R Studio (version 4.0.2) and the package “MendelianRandomization.”

## 3. Results

### 3.1. Causal Associations with PCOS

The *F* statistics of all the selected SNPs exceeded 10 (Table 1), indicating they were not weak instruments.

**Figure 2:**
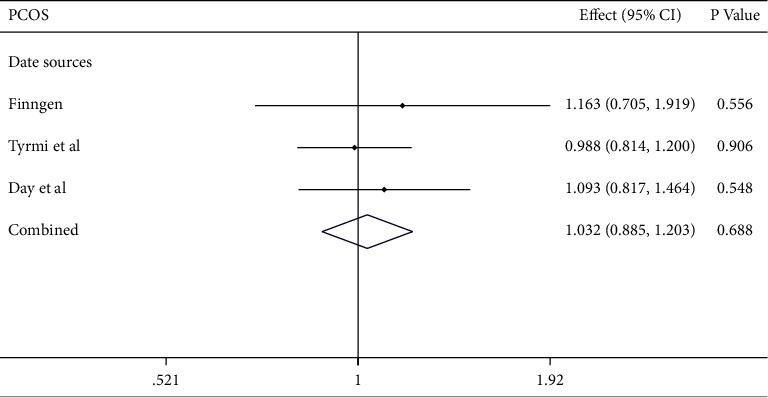
Associations of homocysteine with polycystic ovary syndrome. CI: confidence interval and OR: odds ratio.

**Figure 3:**
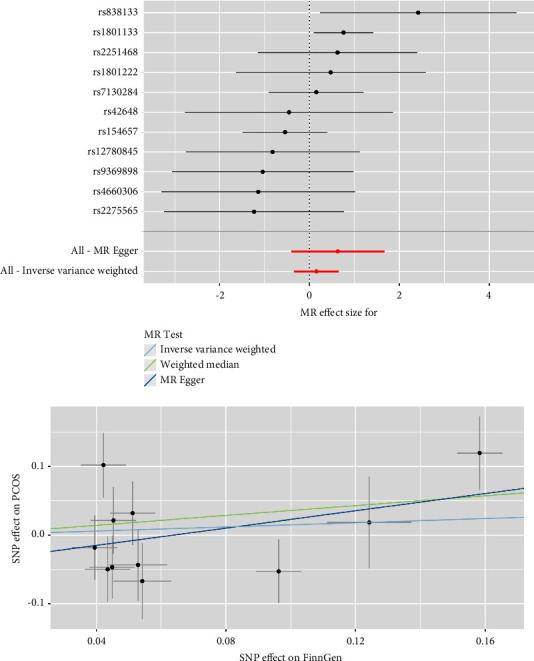
Forest plots and scatter plots for Hcy and PCOS in FinnGen.

**Figure 4:**
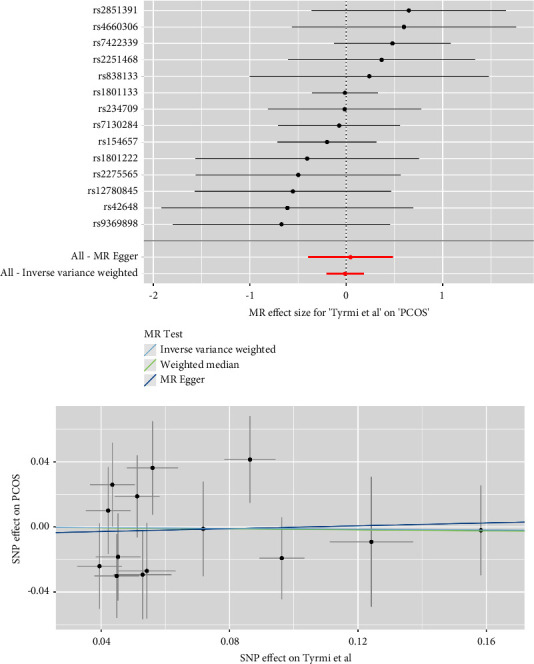
Forest plots and scatter plots for Hcy and PCOS in Tyrmi et al.

**Figure 5:**
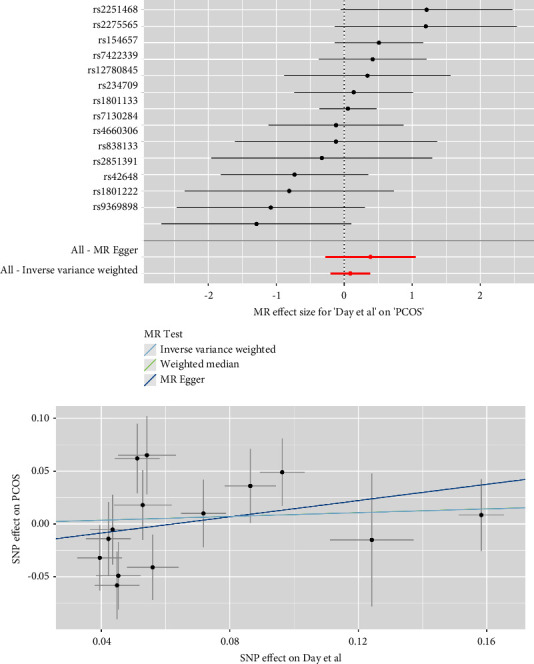
Forest plots and scatter plots for Hcy and PCOS in Day et al.

As depicted in Figure 2, the IVM analyses revealed that plasma Hcy level was not causally associated with the risk of PCOS in the meta-analysis (combined effect = 1.032, 95% confidence interval (CI): 0.885–1.203, *p*=0.688). As anticipated, consistent results were observed in the FinnGen study (effect = 1.163, 95% CI: 0.705–1.919, *p*=0.556), the study of Day et al. (effect = 1.093, 95% CI: 0.817–1.464, *p*=0.548), and that of Tyrmi et al. (effect = 0.988, 95% CI: 0.814–1.200, *p*=0.906). The forest plots and scatter plots for Hcy and PCOS are displayed in Figures 3–5.

### 3.2. Sensitivity Analyses

The results of the weighted median method and MR-Egger regression were in line with the aforementioned findings (Table 2). The MR-Egger intercept analysis, assessing the pleiotropy of the IVW model, delineated no evidence of directional pleiotropy in the FinnGen study (MR-Egger intercept = −0.004, *P*=0.300), the study of Day et al. (MR-Egger intercept = −0.024, *P*=0.332), or that of Tyrmi et al. (MR-Egger intercept = −0.005, *P*=0.780). Pleiotropy tests using MR-PRESSO did not identify any pleiotropic SNPs, suggesting the absence of bias in our study. Taken together, these results suggested that pleiotropy did not influence our estimated association. The MR-Steiger test results supported the absence of reverse causality.

## 4. Discussion

To the best of our knowledge, this is the first study based on MR methods involving adequate sample sizes to examine the correlation between plasma Hcy levels and PCOS, and our result did not identify plasma Hcy levels as a risk factor for PCOS. The selection of homocysteine (Hcy) as a biomarker for assessing the risk of polycystic ovary syndrome (PCOS) as an outcome disease warrants careful consideration of potential confounders. While our study aimed to elucidate the relationship between Hcy levels and PCOS risk, it is imperative to acknowledge the multifactorial nature of PCOS etiology. Factors such as age, body mass index (BMI), hormonal imbalances, insulin resistance, and lifestyle variables can significantly influence both Hcy levels and PCOS development [42]. For instance, higher BMI has been associated with elevated Hcy levels and increased PCOS risk [43]. Furthermore, hormonal imbalances, particularly elevated androgen levels and insulin resistance, are common features of PCOS and may independently impact Hcy metabolism. In addition, dietary factors rich in folate, vitamin B6, and vitamin B12 have been linked to lower Hcy levels and may potentially mitigate the risk of developing PCOS [44]. Therefore, future studies investigating the association between Hcy and PCOS should comprehensively account for these confounding variables to accurately assess the true relationship between Hcy levels and PCOS risk.

Accumulating evidence uncovered these years suggests that higher Hcy levels may play an instrumental role in the etiology of PCOS. A recent meta-analysis, enrolling 1718 PCOS cases and 1399 controls, demonstrated that PCOS patients had statistically significantly higher Hcy levels after individually adjusting for obesity, insulin resistance, and testosterone level [13]. However, heterogeneity was significant in this meta-analysis, and subgroup analyses for these confounders were not performed [13]. Another meta-analysis performed by Murri et al., including 2090 cases and 1421 women without PCOS, found that Hcy levels were 23% higher in PCOS women than in healthy controls [14]. Interestingly, the observed difference between the Hcy levels and PCOS in this study was not significant. This inconsistency may be ascribed to several factors as follows: (1) both studies exhibited considerable heterogeneity, thereby compromising the reliability of their results; (2) publication bias is also an indispensable factor. Publishers are prone to accepting studies with statistically significant results. Negative results regarding the association between Hcy levels and PCOS were identified in limited articles in PubMed and other databases; (3) a large number of past studies have been restrained by small sample sizes, with the biggest meta-analysis merely involving 2090 cases, which is significantly lower than our study population; and (4) Hcy levels are correlated with many confounding variables, encompassing age, BMI, smoking status, concomitant subclinical inflammatory diseases, and insulin resistance [11, 18, 45], which may have influenced the results of the studies. Obesity, a prevalent feature among women with PCOS, has been reported to increase Hcy levels through various pathways, including impaired methylation and increased oxidative stress [46]. Moreover, insulin resistance, a hallmark of PCOS, may contribute to elevated Hcy levels by altering the activity of enzymes involved in Hcy metabolism.

Earlier studies have provided evidence that obesity is a risk factor for PCOS [36]. However, Hcy levels are typically elevated in obese patients [11], indicating that a high-fat diet might aggravate hepatic enzyme activity involved in homocysteine metabolism [47]. While some confounding variables were adjusted in observational studies, they may not fully account for reverse causation. Elevated Hcy levels may stem from various factors associated with obesity, such as dietary patterns and metabolic dysregulation. Indeed, the consumption of high-fat diets among obese individuals exacerbates the activity of hepatic enzymes involved in Hcy metabolism, resulting in elevated Hcy levels [48]. However, it is critical to recognize that altered liver enzyme activity is a direct consequence of obesity. Our study explored their potential impact on diseases such as polycystic ovary syndrome (PCOS), thereby elucidating the complex interplay between metabolic disorders and Hcy metabolism. The robust evidence generated by the MR methodological approach strongly validates this finding.

Nevertheless, some limitations in our study should not be overlooked. First, PCOS patients were not stratified by the abovementioned diagnostic criteria, given that Tyrmi et al. and the FinnGen (R7) study [37, 38] exclusively provided information based on ICD codes without detailed diagnostic information. Besides, the study of Tyrmi et al. contained data from FinnGen data freeze release 6 (R6) and Estonian Biobank, leading to a partial overlap of the outcome data with the FinnGen (R7) study. Second, our study only enrolled individuals of European ancestry to mitigate population structure bias; consequently, our conclusions may not be generalizable to a global population. Finally, PhenoScanner was used to identify potential pleiotropy of the IVs, but the possibility of missing potential pleiotropic effects cannot be excluded, considering that various phenotypes associated with genetic variants are still being explored.

## 5. Conclusion

Our two-sample MR analysis found that plasma Hcy levels were not causally associated with PCOS, which will ultimately help improve prevention and management strategies for this common gynecological condition.

## Figures and Tables

**Table 1:** Characteristic of the instrumental variables (IVs) associated with plasma homocysteine (Hcy) and the associations with polycystic ovarian syndrome (PCOS).

IV	Chr	Nearby gene	Coded allele	Association with Hcy			*F* statistic	Association with PCOS								
				van Meurs et al.				FinnGen			Day et al.			Tyrmi et al.		
				Beta	SE	*P* value		Beta	SE	*P* value	Beta	SE	*P* value	Beta	SE	*P* value
rs1801133	1	MTHFR	A	0.1583	0.007	4.34*E* − 104	511.41	0.1196	0.0536	0.026	0.0085	0.034	0.8	−0.0021	0.0277	0.939
rs2275565	1	MTR	T	−0.0542	0.009	1.96*E* − 10	36.27	0.0670	0.0553	0.226	−0.065	0.037	0.079	0.027	0.0294	0.358
rs7422339	2	CPS1	A	0.0864	0.008	4.58 *E* − 27	116.64	—	—	—	0.036	0.035	0.3	0.0414	0.0267	0.121
rs9369898	6	MUT	A	0.0449	0.007	2.17*E* − 10	41.14	−0.0468	0.0462	0.311	−0.058	0.032	0.067	−0.0301	0.0258	0.242
rs7130284	11	NOX4	T	−0.1242	0.013	1.88*E* − 20	91.28	−0.0186	0.0668	0.780	0.015	0.063	0.82	0.0091	0.04	0.820
rs154657	16	DPEP1	A	0.0963	0.007	1.74*E* − 43	189.26	−0.0526	0.0465	0.258	0.049	0.032	0.12	−0.0192	0.0252	0.448
rs234709	21	CBS	T	−0.0718	0.007	3.90*E* − 24	105.21	—	—	—	−0.01	0.032	0.75	0.0012	0.0291	0.968
rs4660306	1	MMACHC	T	0.0435	0.007	2.33*E* − 9	38.62	−0.0496	0.0478	0.299	−0.0053	0.033	0.87	0.026	0.0258	0.314
rs42648	7	GTPB10	A	−0.0395	0.007	1.97*E* − 8	31.84	0.0181	0.0467	0.698	0.032	0.031	0.3	0.0241	0.0263	0.358
rs1801222	10	CUBN	A	0.0453	0.007	8.43*E* − 10	41.88	0.0214	0.0488	0.662	−0.049	0.032	0.13	−0.0184	0.0268	0.492
rs2251468	12	HNF1A	A	−0.0512	0.007	1.28*E* − 12	53.50	−0.0319	0.0464	0.491	−0.062	0.033	0.058	−0.0188	0.0253	0.458
rs838133	19	FUT2	A	0.0422	0.007	7.48*E* − 9	36.34	0.1021	0.0471	0.030	−0.014	0.035	0.69	0.0101	0.0267	0.706
rs12780845	10	CUBN	A	0.0529	0.009	7.8*E* − 10	34.55	−0.0434	0.0523	0.406	0.018	0.033	0.59	−0.0293	0.0275	0.288
rs2851391	21	CBS	T	0.056	0.008	1.7*E* − 12	49.00	—	—	—	−0.041	0.031	0.19	0.0363	0.0288	0.208

Chr, chromosome; Beta, per allele effect on exposure or outcomes; SE, standard error.

**Table 2:** Weighted median and MR-Egger analysis for genetic associations between exposures and PCOS risk.

Method	Weighted median	MR-egger		MR-PRESSO	Steiger test
		Estimate	Intercept		Direction
*FinnGen*					True
Estimate (95% CI)	0.362 (−0.227, 0.951)	0.630 (−0.406, 1.666)	−0.040 (−0.116, 0.036)		
*P* value	0.228	0.233	0.300	0.118	
*Tyrmi et al.*					True
Estimate (95% CI)	−0.015 (−0.284, 0.253)	0.044 (−0.394, 0.483)	−0.005 (−0.037, 0.028)		
*P* value	0.912	0.843	0.780	0.646	
*Day et al.*					True
Estimate (95% CI)	0.090 (−0.254, 0.434)	0.384 (−0.279, 1.048)	−0.024 (−0.072, 0.024)		
*P* value	0.608	0.256	0.332	0.181	

## Data Availability

All data used to support the findings of this study are available from the corresponding author upon request.
